# Chinese herbal compound prescription for systemic lupus erythematosus: A protocol for systematic review and meta-analysis

**DOI:** 10.1097/MD.0000000000022404

**Published:** 2020-10-02

**Authors:** Yehao Luo, Donghan Xu, Yulei Fu, Xiusong Tang, Zhenfeng Chen, An Huang, Yuzhou Pang, Yunyan Zhang, Renfeng Li

**Affiliations:** a The First Affiliated Hospital of Guangxi University of Chinese Medicine, Nanning, Guangxi Province; b Macau University of Science and Technology, Macau; c Guangxi University of Traditional Chinese Medicine, Nanning, Guangxi Province, China.

**Keywords:** Chinese herbal compound prescription, meta-analysis, protocol, systematic review, systemic lupus erythematosus

## Abstract

**Background:**

Systemic lupus erythematosus (SLE), known as lupus, is a chronic autoimmune disease and there is no cure for SLE. The western medication can improve syndromes to some extent; however, severe adverse drug reactions appear at the same time. Recently, it is confirmed that Chinese medicine also can have an excellent clinical efficacy on SLE.

**Methods and analysis:**

The following databases will be searched for relevant information before July 2020: PubMed, Embase, Cochrane Library, Web of Science, and China National Knowledge Infrastructure. Major results: levels of total remission rate, SLEDAI. Secondary results: The laboratory index about C3 levels, Hb levels, white blood cell levels, and adverse event. Data will be collected independently by 2 researchers, and the risk of bias in meta analysis will be evaluated according to “Cochrane Handbook for Systematic Reviews of Interventions.” All data analysis will be conducted using Review Manager V.5.3. and Stata V.12.0.

**Results:**

The curative effect and safety of Chinese herbal compound prescription treatment for SLE patients will be evaluated systematically.

**Conclusion:**

The systematic review of this study will summarize the currently published evidence of Chinese herbal compound prescription treatment for SLE to further guide its promotion and application.

**Ethics and dissemination:**

The private information from individuals will not be published. This systematic review also will not involve endangering participant rights. Ethical approval is not required. The results may be published in a peer-reviewed journal or disseminated in relevant conferences.

**Open Science Framework (OSF)registration number:**

https://osf.io/wvfrx/.

## 1 Introduction

Systemic lupus erythematosus (SLE), known as lupus, is a chronic autoimmune disease that immune system mistakenly attacks healthy tissue in related organs.^[1]^ Mostly, it occurs in teen female and the classification of symptoms varies from mild cases to severe cases. The cause of SLE is not clearly described yet.^[2]^ However, most researches showed that it is linked to some risk factors, for example, genetics, endocrine dyscrasia, infections, dysimmunity, etc.^[3]^ There is no cure for SLE. Nowadays, the treatment of western medicine involves preventing flares and reducing their severity and duration, which concludes medications such as prednisone, mycophenolic-acid and tacrolimus, disease-modifying antirheumatic drugs, immunosuppressive drugs, and analgesia.^[4]^ Nevertheless, the medications may cause or burden the impair of other organs and secondary infection with low immunologic function.

Certain studies have found that SLE is the main cause of death in rheumatic diseases. So far, the global average prevalence of SLE is 12–39/100 thousand, and 30–70/100 thousand in China, which takes up the second place in the world. Besides, the mean age of onset is about 29 years old for SLE in China. The first clinical manifestation was hematologic abnormality (56.1%), arthritis (54.5%), butterfly erythema (47.9%), nephrosis (47.4%), and fever (37.8%) in order.[^5^,^6^]

Recently, more studies expressed that during long term clinical practices of Chinese medicine, therapists had a pronounced understanding of pathogenesis of SLE and the treatment of Chinese herbal compound prescription achieved a great progress on SLE.^[7]^ However, there is a lack of evidence of results of combination of Chinese and western medicine in treating SLE. Therefore, the paper will evaluate the effectiveness and safety of Chinese herbal compound prescription treatment for SLE. This review will be the first evaluation of the impact of Chinese herbal compound prescription treatment.

## 2 Objectives

In a randomized controlled trial (RCT), the efficacy and side effects of Chinese herbal compound prescription in treating SLE have been evaluated systematically. We expect to provide reference for SLE treatment in the field of traditional Chinese medicine.

## 3 Methods

### 3.1 Study registration

The protocol of the systematic review has been registered.

Registration: OSF Preregisration.2020, Aug.22. osf.io/wvfrx/. This systematic review protocol will be conducted and reported strictly according to Preferred Reporting Items for Systematic Reviews and Meta-Analyses (PRISMA)^[8]^ statement guidelines, and the important protocol amendments will be documented in the full review.

### 3.2 Inclusion and exclusion criteria for study selection

#### 3.2.1 Inclusion criteria

Inclusion criteria are all randomized controlled trials (RCTs), which main treatment of SLE is Chinese herbal compound. The language of the trials to be included is only Chinese or English.

#### 3.2.2 Exclusion criteria: Following studies will be excluded

1. Repeated publications
2. Review of literature and cases
3. Animal studies
4. Incomplete literature
5. Nonrandomized controlled trials

### 3.3 Types of participants

We will include RCTs of participants of 18 years or older, of any sex, race/ethnicity.

Systemic lupus erysiatosus activity index score within this range: 5 ≤ SLEDAI ≤ 14, C ≤ BILAG ≤ A. We will exclude except hydroxychloroquine and glucocorticoid, combined use of other immunosuppressants in recent 1 month. Patients with other connective tissue diseases, severe lupus, drug-induced lupus syndrome, and tuberculosis; Patients with severe system injuries, tumors, psychosis, etc. caused by non-SLE; Female patients during pregnancy or lactation; Allergic to the ingredients contained in the study drugs; Failure to use the medicine as prescribed, failure to evaluate the curative effect and incomplete data.

### 3.4 Interventions and controls

Interventions included treatment with Chinese herbal compound. The control group only received conventional western medicine treatment. The routine treatment of each RCT may not be identical, but the use of Chinese herbal compound is the only difference between intervention and control.

### 3.5 Type of outcome measures

#### 3.5.1 Main outcomes

1. total remission rate;
2. SLE DAI

#### 3.5.2 Additional outcomes

1. C3 levels;
2. Hb levels;
3. white blood cell levels;
4. adverse events.

### 3.6 Search methods

#### 3.6.1 Search resources

**Figure 1:**
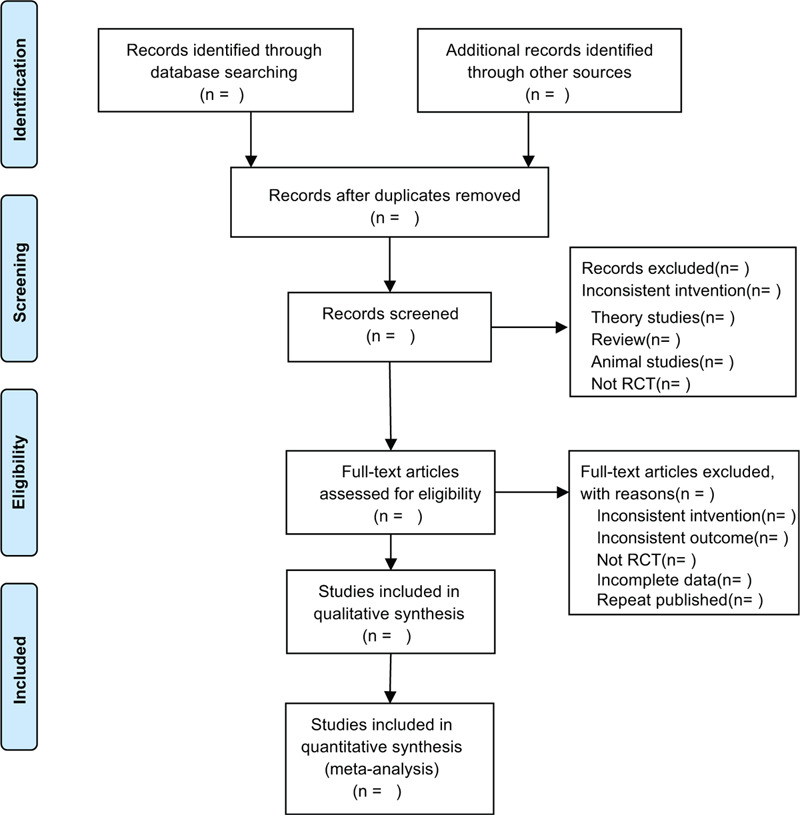
The research flowchart. This figure shows the Identification, Screening, Eligibility, and Included when we search articles.

This review will include the following electronic databases from their inception to July 2020: Electronic database includes PubMed, Embase, Cochrane Library, Web of Science, China National Knowledge Infrastructure (Fig. 1). The research flowchart.

#### 3.6.2 Search strategies

The following MeSH terms and their combinations will be searched: Systemic lupus erythematosus OR SLE; RCT OR RCTs; Preparation of traditional Chinese medicine OR Chinese herbal compound OR Chinese herbal medicine compound preparation OR Chinese herbal compound prescription OR Traditional medicine compound. The search strategy for PubMed is shown in Table 1. Other electronic databases will use the same strategy.

**Table 1:**
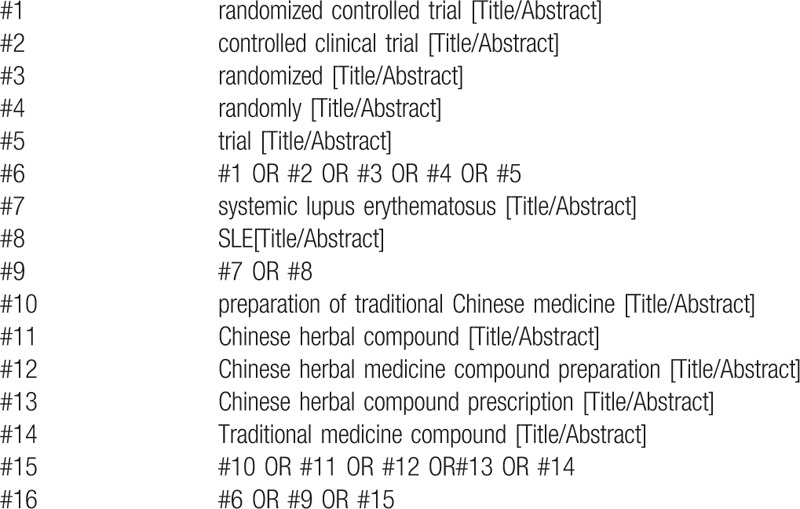
Search strategy in PubMed database.

### 3.7 Data collection and analysis

#### 3.7.1 Studies selection

There will be 2 researchers (YL and DX) carrying out the selection of research literature independently using endnote x9 software. We will first make the preliminary selection by screening titles and abstracts. Secondly, we will download full text of the relevant studies for further selection according to the inclusion criteria. If there is any different opinion, 2 researchers will discuss and reach an agreement. If a consensus could not be reached, there will be a third researcher (GF) who will make the final decision. The details of selection process will be displayed in the PRISMA flow chart.

#### 3.7.2 Assessment of risk of bias

The assessment of risk of bias will be carried out by 2 independent reviewers (YL and ZC), using the Cochrane Collaboration's “Risk of bias” tool. Study bias will be conducted as either: “unclear,” “low,” or “high” risk for the following criteria: random sequence generation, allocation concealment, blinding, incomplete data, selective outcome reporting, and other bias. The assessment of the bias has caused controversy, there is a need for discussion with a third reviewer (RL). The graphic representations of potential bias within and across studies using Rev Man V.5.3.5.

#### 3.7.3 Measures of treatment effect

Statistical analyses will be conducted using the risk ratio with 95% confidence intervals (CIs). Odds ratio (OR) and relative risk (RR) are commonly used for dichotomous outcomes data. For continuous outcomes, the weighted mean difference or the standard mean difference will be analyzed.

#### 3.7.4 Unit of analysis issues

The unit of analysis will be the individual participant.

#### 3.7.5 Dealing with missing data

Among the results of several studies with insufficient data or missing data, the corresponding author will be contacted to complement the contents. If the corresponding author cannot be contacted, the data alone will be conducted.

#### 3.7.6 Assessment of heterogeneity

The assessment of heterogeneity will be conducted by Review Manager (V.5.3.5). Chi-squared test and I2value of the forest, plot will be calculated to assess heterogeneity, according to the Cochrane Handbook. The I2 value is classified into 4 levels: little or no heterogeneity (0%–40%), moderate heterogeneity (30%–60%), substantial heterogeneity (50%–90%), and considerable heterogeneity (75%–100%).

#### 3.7.7 Assessment of reporting biases

If the numbers of available studies are sufficient, funnel plots will assess reporting biases.

#### 3.7.8 Data synthesis

Review Manager (V.5.3.5) will be used to analyze. The test indicated little or no heterogeneity; a fixed effect model will be used for data. The random effect model will be adopted when there is considerable heterogeneity (I2 ≥ 50%). If there is considerable variation in results (I2 ≥ 75%), the meta-analysis will not be performed. The narrative and qualitative summary will be available.

#### 3.7.9 Subgroup analysis and investigation of heterogeneity

Subgroup analysis will be conducted to assess heterogeneity. The different types of Chinese herbal compound prescription (“Yiqi Bushen Huoxue decoction,” “Fuzheng Jiedu decoction,” “Yiqi Yangxue Jianpi Bushen decoction,” or “Liu Wei Di Huang Wan”) may affect heterogeneity.

#### 3.7.10 Sensitivity analysis

Sensitivity analysis will be used to assess the robustness of the results. It is possible to determine according to methodological quality, sample size, and analysis-related issues. The studies that follow a sequence will be removed from all the inclusion reviews. The chi-squared test and I2 value will be used to quantify statistical heterogeneity.

#### 3.7.11 Summary of evidence

The assessment of evidence for all outcomes will be summarized using the Grading of Recommendations Assessment, Development and Evaluation (GRADE) approach. The quality of evidence will be rated as high, moderate, low, and very low quality.

## 4 Discussion

Systemic lupus erythematosus (SLE) is a chronic multisystem autoimmune disease of unclear etiology. The course of 4disease is complex.^[9]^ At present, Western Medicine mainly treats with glucocorticoid and immunosuppressants in order to alleviate the state of disease; however, some side effects such as bone marrow suppression, liver and kidney damage, and osteoporosis could be one of the causes of SLE mortality.^[10]^ As an important part of traditional medicine, traditional Chinese Medicine (TCM) is one of the effective means of comprehensive treatment of SLE, which can effectively reduce the side effects caused by Western medicine. Most scholars consider that SLE should be treated with the treatments of combination of traditional Chinese medicine and Western medicine.^[11]^ Besides, treatments based on syndrome differentiation of TCM can play the advantages in different stages during the treatment of long-term SLE, so as to achieve the sustainability and safety of treatment. So far, the treatments of combination of TCM and Western Medicine applied in SLE have been widely reported, and the clinical efficiency is remarkable. For example, a study^[12]^ found that systemic treatment of SLE with TCM and Western Medicine can increase the effective rate to 86.0%, moreover, improve symptoms such as fever, Erythema, and joint pain during treatment, and reduce adverse reactions. The research^[13]^ demonstrates that hormone combined with TCM treated in SLE can not only relieve the symptoms as soon as possible, but also control the development of the disease.

However, the mechanism and standards of treating SLE using Chinese herbal compound prescription are not expounded systematically. In short, this systematic review and meta-analysis can help identify the potential value of Chinese herbal compound prescription in treating SLE and improving the clinical symptoms, related laboratory indexes apparently and life quality. This study can provide a foundation for the release of SLE treatment guidelines and treatment options of SLE patients, and thus benefit more patients.

## Author contributions

**Conceptualization:** Yehao Luo, Donghan Xu.

**Data curation:** Donghan Xu, Yulei Fu, Fang Gang.

**Formal analysis:** Yehao Luo, Xiusong Tang, Zhenfeng Chen.

**Funding acquisition:** Yuzhou Pang, Fang Gang, Renfeng Li.

**Investigation:** Donghan Xu, Yulei Fu, Yunyan Zhang.

**Methodology:** Yulei Fu, Yuzhou Pang.

**Project administration:** Yuzhou Pang, Renfeng Li.

**Quality assessment:** Fang Gang, Yehao Luo, Yunyan Zhang.

**Software:** Zhenfeng Chen, Donghan Xu, An Huang, Xiusong Tang.

**Supervision:** Yehao Luo, Renfeng Li, An Huang.

**Validation:** Zhenfeng Chen, Yunyan Zhang, Yehao Luo, An Huang.

**Writing – original draft:** Yehao Luo, Donghan Xu, Xiusong Tang.

**Writing – review & editing:** Donghan Xu, Fang Gang, Yuzhou Pang, An Huang, Renfeng Li.
